# Loneliness of Older Adults: Social Network and the Living Environment

**DOI:** 10.3390/ijerph16030406

**Published:** 2019-01-31

**Authors:** Astrid Kemperman, Pauline van den Berg, Minou Weijs-Perrée, Kevin Uijtdewillegen

**Affiliations:** Department of the Built Environment, Eindhoven University of Technology, 5600MB Eindhoven, The Netherlands; a.d.a.m.kemperman@tue.nl (A.K.); m.weijs.perree@tue.nl (M.W.-P.); kevin_uytdewillegen@hotmail.com (K.U.)

**Keywords:** loneliness, aging, social network, social participation, neighborhood, Bayesian belief network (BBN)

## Abstract

The social participation and integration of older adults are important aspects of healthy aging. However, in general, older adults have smaller social networks than their younger counterparts due to changes in their life cycle stage, such as retirement or age-related losses, along with a declining health and increasing mobility limitations. Consequently, with increasing age, an increasing proportion of older people experience feelings of loneliness and social isolation. Previous studies that have analyzed the relationships between loneliness, social networks, and the living environment have often been based on bivariate relationships or included only a limited number of variables. Therefore, the aim of this study was to analyze multiple relationships in a more comprehensive framework. Data were collected using a survey among 182 adults aged 65 years and over in the Netherlands. A Bayesian belief network (BBN) modeling approach was used that derives all direct and indirect relationships between the variables. The results showed that feelings of loneliness are directly related to satisfaction with one’s social network and neighborhood attachment and are indirectly related to perceived safety and satisfaction with local amenities and services. This knowledge is relevant to urban planners and policy makers who focus on creating livable and healthy social neighborhoods for the aging population.

## 1. Introduction

Like that of other countries worldwide, the population of the Netherlands is increasingly aging. The group of people aged 60 years and over is increasing faster than younger age groups [1]. In 2040, it is expected that a quarter of the Dutch population will be 65 years and over [2].

Compared to previous generations, older adults are generally more active, healthier, wealthier, and higher educated [3]. On the other hand, older adults are more often single and childless. In addition, due to changes in their life cycle stages, such as retirement or age-related losses (e.g., death of a partner or friends), along with a declining health and increasing mobility limitations, an increasing percentage of older adults experiences feelings of loneliness and social isolation [4,5,6]. Therefore, interest in subjective aspects of the quality of life of older adults, such as well-being, happiness, social satisfaction, and loneliness, is increasing [7,8,9,10]. The social participation and integration of older adults appear to be important aspects of healthy aging, as they are positively associated with quality of life [11,12]. 

Older adults mainly prefer to remain in their own house and live independently [13]. In addition, policy makers and health care companies increasingly promote aging in place [14], which refers to “remaining living in the community, with some level of independence, rather than in residential care” ([15], p. 133). For aging in place, older adults are more dependent of their own social network (e.g., family and friends) [16,17]. As older adults spend more time in the neighborhood and stay longer in the same living environment than younger (working) adults [18], neighborhood contacts are important for stimulating social inclusion, place attachment, and social satisfaction [19,20]. In addition to the social structure of the living environment, previous studies also showed relationships between the physical living environment (e.g., type of housing, urban density, and amenities), social participation, and eventually feelings of loneliness [11,21,22]. Furthermore, evidence for the effects of sociodemographic and mobility characteristics on older adults’ feelings of loneliness has been found [23,24,25]. 

Although previous studies observed factors that explain differences in social participation and loneliness, such as personal characteristics, mobility, and the social and physical living environment, it remains a major challenge to create livable and healthy social neighborhoods for the aging population that prevent older adults from social isolation and feelings of loneliness. Therefore, aging of the population is still a widely discussed subject by governments, municipalities, social housing associations, and health care companies. Existing knowledge has often been based on bivariate relationships between these factors or studies only including a limited number of variables. Thus, the aim of this study was to bring all these factors together into a more comprehensive framework to explain the (direct and indirect) relationships between sociodemographics, living environmental characteristics, social participation, and loneliness.

Data were collected by a survey among 182 adults aged 65 years and over living in the West Brabant region in the Netherlands. For the analysis, a Bayesian belief network (BBN) modeling approach was used that derives and represents all direct and indirect relationships between the variables. The results of this study could help urban planners and policy makers who focus on creating livable and healthy social neighborhoods for the aging population. The remainder of this paper is structured as follows. First, based on a literature review, possible relationships were identified between sociodemographics, social network characteristics, mobility characteristics, and characteristics of the living environment. The next section describes the data collection procedure, the sample, and modeling approach, followed by the results. The paper ends with a discussion of the results, conclusion, and limitations.

## 2. Literature Review

Loneliness can be defined as “the subjective evaluation of the situation individuals are involved in, characterized either by a number of relationships with friends and colleagues which is smaller than is considered desirable (social loneliness), as well as situations where the intimacy in confidant relationships one wishes for has not been realized (emotional loneliness)” ([26], p. 121). It is recognized that loneliness is influenced by the size of people’s social network [27,28]. People who have fewer people in their social network are more likely to feel lonely than people who have a large social network [29]. Furthermore, research showed that the composition of the social network is also an important indicator for loneliness. People who have a family-dependent support network (i.e., close family ties with few neighborhood and friend links) and people who have a private restricted support network (i.e., no relatives, few nearby friends, and low levels of community involvement) are more at risk of feeling lonely [30,31]. Other studies found a relation between being satisfied with the social network and loneliness [32]. However, a lower number of social interactions does not necessarily mean that people feel lonelier or are less satisfied with their social network [11,33].

Previous studies found that older adults have fewer social interactions and a smaller social network than younger adults [5,32]. This decrease of the social network size is mainly due to life events, such as retirement or the loss of family members, friends, and neighbors [34]. 

An earlier study showed that, based on a meta-analysis of 102 studies on loneliness, women report significantly higher levels of loneliness than men [35]. Further, people with poorer health conditions tend to feel lonelier [26,36,37]. When people get older, they, in general, are less mobile and have more limited activity spaces [38], so they will probably feel lonelier than younger adults. In addition, older people tend to feel less lonely if they live with a partner and if they have (more) children [26,28]. Furthermore, studies showed that a higher income, contact with children, and nonprofessional activities could positively affect social satisfaction [9,33]. People with a low education level and a low-income level have been found to be more likely to feel lonely [28,35].

In addition to sociodemographic characteristics, studies also found evidence for the influence of mobility characteristics (e.g., frequency of using different transport modes and ability to perform activities) on feelings of loneliness. Mobility becomes increasingly important as it provides access to social interactions that are important for the social and emotional well-being of people [39,40]. Research, for example, showed that the use of different transport modes (e.g., bicycle, car, and public transport) and car ownership are significantly related to lower levels of loneliness [25,32]. In addition, frequently walking in the neighborhood could lead to more spontaneous social interactions [41], which could in turn reduce loneliness. With regard to the ability to perform activities, previous research showed that people who are fully dependent while performing daily activities have higher feelings of loneliness [42].

Having infrequent neighborhood contacts is related to higher levels of loneliness [43]. Older adults are probably more dependent on their social network in the neighborhood than younger adults, as they are more likely to have lived a long time in the same neighborhood and to spend more time in this neighborhood. Furthermore, people who know more people within their local area and are more attached to their living environment are less likely to feel lonely [32,44]. Previous studies also found a relation between neighborhood safety and older adults’ mental well-being [45].

With regard to the physical characteristics of the living environment, Kearns et al. [46] found relationships between residents’ perception of the neighborhood physical quality (i.e., attractiveness of buildings and the area, quiet and peacefulness of the area, quality of parks and open spaces, street lighting, and paths and pavements), the use of neighborhood amenities, and loneliness. Another study showed that people living in a highly urbanized and deprived neighborhood are lonelier [24]. Density and length of residence are also predictors of social satisfaction, which is negatively related to loneliness [11].

**Figure 1:**
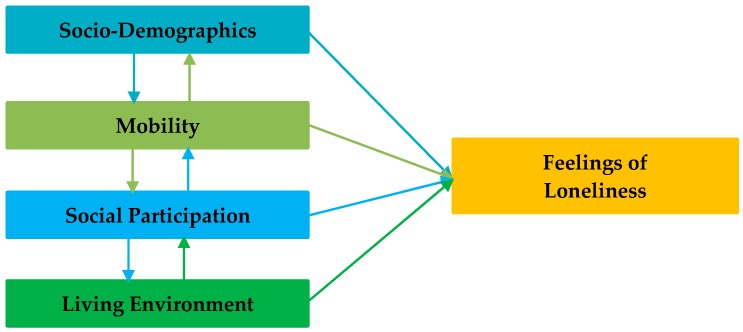
Conceptual model: Factors influencing loneliness in the aging population.

To summarize, the following factors are assumed to influence feelings of loneliness and isolation: Personal and household sociodemographics, individual mobility characteristics, social network and participation, and characteristics of the living environment. See Figure 1 for a conceptual model including these factors.

## 3. Materials and Methods 

### 3.1. Data Collection

The aim of this study was to bring all the factors as discussed in the literature review and shown in the conceptual model (Figure 1) together into a more comprehensive framework to explain the (direct and indirect) relationships between sociodemographics, mobility level, characteristics of the living environment, social participation, and feelings of loneliness among the aging population. To test the conceptual model, data were collected among adults aged 65 years and over living in the West Brabant region in the Netherlands. In the fall of 2015, 400 older adults were personally approached in 17 municipalities of West Brabant, based on convenience sampling. A total of 216 older adults accepted to participate in this study and filled in a paper-and-pencil survey. This eventually resulted in 182 complete and useable surveys. The survey included a variety of questions measuring all factors from the model. All respondents gave their informed consent for inclusion before they participated in the study. The study was conducted in accordance with the Declaration of Helsinki. The questions were asked in Dutch but were translated for the purpose of this manuscript. 

### 3.2. Measures

#### 3.2.1. Sociodemographics

In the survey, questions were asked about personal and household characteristics: Age, income level (low < €1200 net per month; medium = €1200–2400; high > €2400), gender, and whether a respondent (still) has a partner or not. The respondents’ own perceived health status was asked in 5 categories from very bad to very good.

#### 3.2.2. Mobility

Mobility intensity was operationalized by asking how often the respondents use a particular type of transport mode (car driver, car passenger, train, bus, moped, (e-)bike, walking, taxi, scoot mobile) on a 7-point scale from 1 (hardly ever) to 7 (almost every day). Based on the answers about transport mode usage, a principal component analysis with varimax rotation was performed (see Table 1) on the 3 most often used transport modes: Car driver, bicycling, and walking. The main reason for using principal component analysis was to reduce the number of factors for the Bayesian network analysis, while maintaining the most important information. Varimax rotation is an orthogonal rotation, assuming no correlations between components and preserving an essential property of the principal component analysis. Individual factor scores were calculated and used in the remainder part of the analysis. The analysis resulted in 1 factor describing the general mobility intensity. 

The Groningen activity restriction scale (GARS) [47] was used to measure a respondents’ ability or inability to perform activities of daily living (ADL). For 18 activities (dress yourself, get in and out of bed, stand up from sitting in a chair, wash your face and hands, wash and dry your whole body, get on and off the toilet, feed yourself, get around in the house, go up and down the stairs, walk outdoors, take care of your feet and toenails, prepare breakfast or lunch, prepare dinner, do “light” household activities, do “heavy” household activities, wash and iron your clothes, make the beds, and do the shopping), the respondents indicated whether they can fully independently perform these activities on a scale from 1 (need complete help) to 4 (without any difficulty). The answers given were summed per respondent, resulting in scores ranging from 22 indicating a very low to 72 a very high level of independence in performance of activities of daily living. 

#### 3.2.3. Social Participation

To describe social participation, first the size of the respondents’ social network was measured by asking them to list all contacts that support them in either an emotional (e.g., caring, esteem, etc.), instrumental (e.g., informational, tangible), or social (e.g., talk, social visit) way. The network sizes ranged from 0 to 19 persons.

Secondly, the satisfaction with their social contacts, with their social network size, and with the quality of their social network were measured on a 5-point scale ranging from very unsatisfied to very satisfied. Principal component analysis with varimax rotation was conducted on these three items. As can be seen in Table 2, the analysis identified one factor describing the satisfaction with one’s social network.

Thirdly, the respondents indicated where and how often (on a 7-point scale from almost never to daily) they have social contacts at specific locations in their neighborhood (see Table 3 for the locations). Principal component analysis with varimax rotation was used and resulted in 3 factors describing the locations where people have social contacts in their neighborhood: Recreation spaces, community amenities, and shopping amenities.

#### 3.2.4. Living Environment

To define the characteristics of the living environment influencing feelings of loneliness, a number of questions was included in the survey. The neighborhood attachment was asked by a 7-item scale (see Table 4), and for each item, the respondents indicated whether they agreed or disagreed with the statements on a 5-point scale (1 completely disagree to 5 completely degree) [48]. A principal component analysis with varimax rotation resulted in 1 factor describing neighborhood attachment.

Respondents were asked to indicate their feelings of safety in the neighborhood both during the day and at night on a 5-point scale. Principal component analysis showed that the answers on these questions can be combined into 1 factor describing feelings of safety in the neighborhood, see Table 5.

Further, the respondents were asked to indicate their satisfaction with a variety of amenities in their neighborhood on a 5-point scale from very unsatisfied to very satisfied. A principal component analysis with varimax rotation resulted in 1 factor describing the satisfaction with amenities in the neighborhood, see Table 6.

Objective physical environmental characteristics were derived from Statistics Netherlands (CBS) and merged with the survey data based on the four-digit postal code of the home addresses of the respondents. Specifically, distances to public green and to daily shopping amenities were included as variables. Furthermore, the degree of urbanization was measured based on the average number of addresses per 500-meter square within a kilometer radius from the address. This indicator has been widely used in the Netherlands and consists of five categories ranging from very strongly urbanized to not urbanized, rural areas.

#### 3.2.5. Loneliness

Finally, feelings of loneliness were measured using the 6-item loneliness scale [49]: I experience a general sense of emptiness; There are plenty of people I can rely on when I have problems; There are many people I can trust completely; I miss having people around; There are enough people I feel close to; and I often feel rejected. They answered to the statements on a five-category scale ranging from (1) Yes! To (5) No! Following the authors, sum scores were used to measure the overall level of feeling of loneliness. The resulting scores showed a normal distribution ranging from 6, indicating that someone feels not lonely at all, to 26, indicating that there is a very strong feeling of loneliness.

### 3.3. Bayesian Belief Network Modeling

A Bayesian belief network (BBN) was used to formulate and estimate the direct and indirect relationships and their directions between the variables as described in previous section and shown in Table 7 [50,51,52]. Including a large number of variables in the model and finding meaningful relationships was quite a challenge [53]. The structure of the relationships is typically not clear (e.g., mediating effects, interaction effects), and variables are often correlated. This makes the variable selection and finding an appropriate structure for explanatory variables typically difficult. A BBN approach can overcome such difficulties, as it derives and represents simultaneously all direct and indirect relationships between the set of variables. Note that all variables included in the BBN estimation are discretized in categories. A BBN model can, in contrast to, for example, structural equation modeling, deal with discrete variables [50]. This is an advantage, as a number of variables included in the model are of a discrete nature (e.g., gender, household type).

Formally, a BBN is a directed acyclic graph composed of a set of variables that are connected by links to indicate their dependencies:

BBN = (V, E)(1)

where V is a set of variables X, Y, …, and E is a set of links (X,Y).

The links contain information about the causal or temporal relationships between the variables and are represented by arrows. When there is a link between X→Y, X is called a parent of Y, and Y a child of X (of course, Y could be the parent of another variable).

Estimating a BBN from data involves firstly learning the network structure and secondly finding the conditional probability (CP) tables for each variable. BBN learning is based on the three-phase dependency method that develops the network based on tests of conditional independencies between pairs of variables [54]. Secondly, based on the commonly used expectation-maximization (EM) learning algorithm, the CP tables are estimated [55]. This algorithm tries to find the conditional probability distributions and works by an iterative process; it starts with a candidate BBN and uses it to find a better one by doing an expectation (E) step. This is followed by a maximization step (M). This process is repeated until the log likelihood numbers are no longer improving (according to a tolerance that is specified). The CP tables express the probabilities for a variable, conditioned on the values of its parent variables (if any), and are referred to as the parameters of the network. The program Genie [56] was used to learn the network structure and estimate the CP tables.

Before constructing the BBN, the researcher can define constraints on the presence of links between variables and predefine special cases for the network structure a priori based on (expert) knowledge. Specifically, links can be forced (these links are guaranteed to appear in the learned structure) or forbidden (these links are guaranteed to be absent in the learned structure), and variables can be assigned to temporal tiers. The latter means that in the resulting network, there will be no link from variables that occur later in time (in higher tiers) to variables happening earlier in time (in lower tiers). For the construction of our BBN, we tried to limit the number of constraints to find the best fitting model based on the data. However, we put the variables in temporal tiers based on logic; for example, gender and age were put in the first tier as they, by nature, cannot be influenced by any other variables.

In this study, the direct and indirect influence of sociodemographics, mobility intensity, characteristics of the living environment and social participation on feelings of loneliness among the aging population was explored and predicted. Table 7 gives an overview of all the variables and their categories that were included in the model estimation.

After constructing a BBN, it may be applied to a particular case. For example, the effect of changes in neighborhood attachment on feelings of loneliness can be predicted. For some variables, values can be entered as a finding, and the probabilistic changes in other variables can be predicted and changes under certain conditions can be simulated. When new findings are entered into the network, the CP tables of all variables can be updated based on probabilistic reasoning methods that are based on the Bayesian method of belief updating.

## 4. Results

### 4.1. Profile of Respondents

The profile of the respondents is described by the sociodemographic variables in Table 7. The results showed that slightly more women than men participated in the study. They were almost equally distributed over the age categories from 65 to 86+ years of age. About 40% of the respondents were found to have a partner. Over 40% of the respondents perceived their own health as good or very good, while over 21% indicated their health as bad or very bad. Finally, with regard to income level, most respondents (81.3%) indicated that they are in the medium income level category.

### 4.2. Bayesian Belief Network

**Figure 2:**
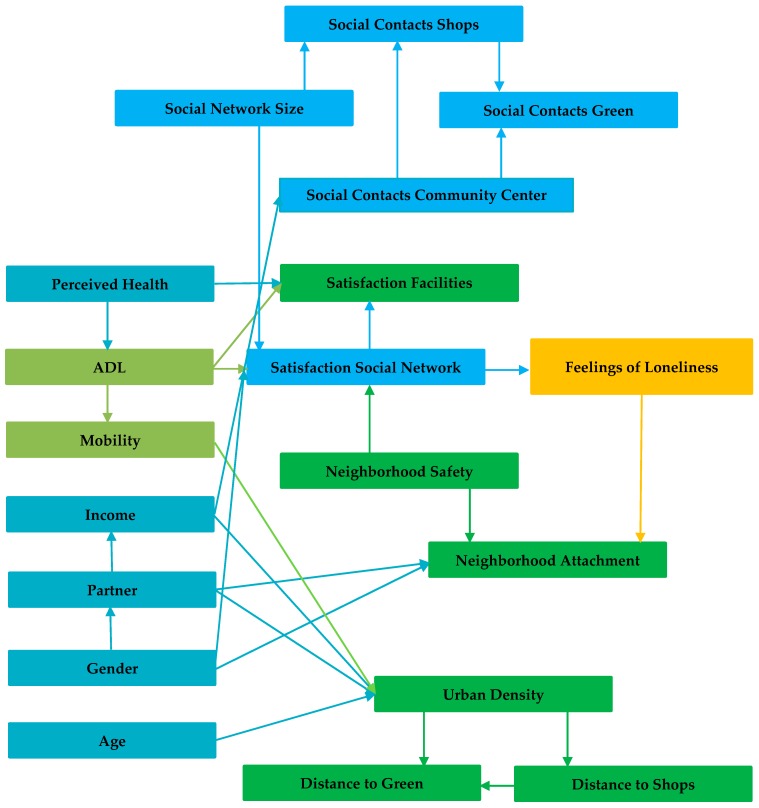
Bayesian belief network model. ADL: activities of daily living.

**Figure 3:**
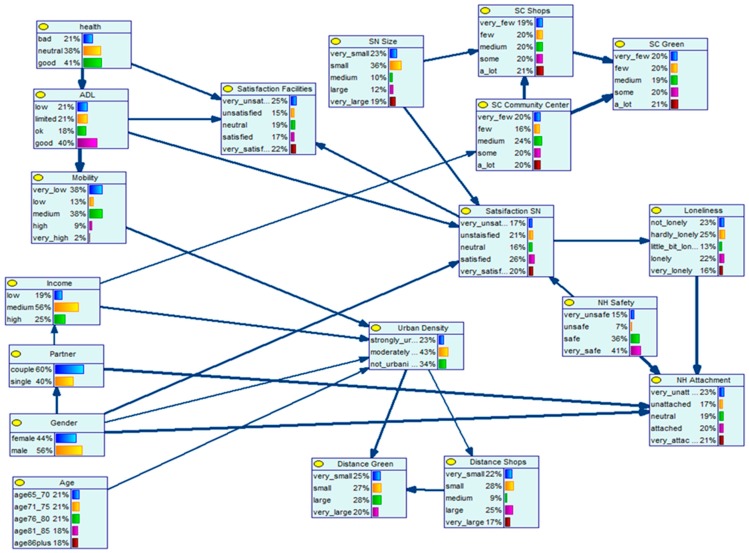
Bayesian belief network model with conditional probability tables. SN: Social Network, SC: Social Contacts, NH: Neighborhood.

For estimating the Bayesian belief network (BBN) structure, the database including all variables as described in Table 7 was taken as the input and the belief network structure was constructed as the output. First, for reasons of clarity, the resulting network structure is presented in Figure 2 and secondly, the structure including the conditional probability (CP) tables for each variable is shown in Figure 3. The network presented in Figure 3 includes for each variable the probability distribution across the categories of that variable, based on the relationships with the parent variable(s). The arrows represent the relationships between two variables.

Firstly, the network structure shows a more complex model structure than the proposed conceptual model (Figure 1). Loneliness was directly influenced by people’s satisfaction with their social network, but feelings of loneliness and isolation were also directly related to the attachment with the neighborhood. Some indirect effects were also interesting. Both the attachment to the neighborhood and satisfaction with the social network were influenced by feelings of safety in the neighborhood. Moreover, the satisfaction with the social network was related to the satisfaction with the amenities in the neighborhood and a person’s ability to perform activities of daily living. The latter variable also directly influenced the satisfaction with the amenities available in the neighborhood. 

Secondly, Figure 3 shows the BBN network, including the CP tables. These tables are presented in bar diagrams at each node and show the probability distribution across the categories of the variables. This model can be used to predict the effect of one variable on one or more other variables in the network. Specific evidence can be entered for one variable in the network structure (for example setting one category of a variable at 100%) and, subsequently, the probabilities of the other variables can be updated. The most important relationships as shown in the network are discussed in detail in the following sections.

#### 4.2.1. Satisfaction with Social Network and Feelings of Loneliness

The BBN model showed that feelings of loneliness and isolation among the aging population are directly influenced by the satisfaction with one’s social network. Table 8 shows the updated probabilities in percentages for the various categories of satisfaction with the social network. For example, assume that all people (100%) are very unsatisfied with their social network, then 0% feels not lonely, 7% feels hardly lonely, and 7% feels a little bit lonely, while 30% feels lonely and 56% feels very lonely. On the other hand, when all people are satisfied with their social network (100% very satisfied), 53% feels not lonely, 33% feels hardly lonely, 7% feels a little bit lonely, also 7% feels lonely, and 0% feels very lonely. Note that because the percentages are rounded, they might not exactly sum up to 100%. The results clearly indicated that people who were very unsatisfied about their social network had a high chance to feel very lonely. Conversely, people who were very satisfied about their social network felt in general not or not so lonely.

#### 4.2.2. Neighborhood Attachment and Feelings of Loneliness

Whether an aging person feels lonely was also directly related to his or her attachment to the neighborhood. As shown in Table 9, people who felt less lonely were in general more attached to their neighborhood and conversely, of the people who feel not or hardly lonely, 54 percent felt very attached to their neighborhood. 

#### 4.2.3. Health Status and Satisfaction with Amenities in the Neighborhood

A person’s perceived own health and the ability to participate independently in activities of daily living both influenced the satisfaction with the amenities available in the neighborhood. In Table 10, the updated probabilities for satisfaction with the amenities in the neighborhood are presented for the level of independence in participating in activities of daily living. Note that the relationship with health showed a similar pattern. In general, the findings showed that people with a lower level of independence in activities of daily living were less satisfied with the amenities provided in the neighborhood, while they were more satisfied if they were better able to perform these activities independently. Moreover, aging persons who were more able to perform all kinds of activities for daily living were in general more mobile than persons who needed support in performing these daily activities.

#### 4.2.4. Neighborhood Safety and Neighborhood Attachment 

The level of perceived safety in the neighborhood had a significant influence on the attachment with the neighborhood. As indicated in Table 11, aging persons who felt unsafe in their neighborhood also felt less attached to their neighborhood and those who felt very safe in the neighborhood also felt much more attached to their neighborhood.

#### 4.2.5. Neighborhood Safety and Satisfaction with Social Network

Perceived safety in the neighborhood was also related to someone’s satisfaction with his or her social network. The higher the perceived level of safety, the more satisfied with their network and conversely, a low level of perceived safety was in line with a lower satisfaction with the social network. The updated probabilities for the satisfaction with the social network based on the perceived level of safety on the neighborhood are presented in Table 12.

## 5. Discussion

The aim of this study was to investigate the relationships between sociodemographics, characteristics of the living environment, social participation, and loneliness of older adults. The results showed that only the satisfaction of older adults with their social network was directly related to feelings of loneliness, which was also confirmed by a previous study on loneliness [32]. Previous research also found a direct relation between social network size and loneliness [29,43]. However, this current study only found an indirect relation, namely, the results showed that social satisfaction played a mediating role in the relation between social network size and feelings of loneliness. Thus, people who had a larger social network were more likely to be satisfied with their social network and subsequently less likely to feel lonely.

With regard to the living environment, neighborhood safety was also found to be an important predictor for social satisfaction and neighborhood attachment. Another study also found that neighborhood safety and social problems in the neighborhood were more important predictors of neighborhood satisfaction than the physical characteristics of the neighborhood [57]. In addition, the satisfaction with amenities in the neighborhood was found to be related to social satisfaction and, indirectly, to loneliness. This indicates the importance of high-quality amenities. Policy makers should thus focus on creating more safe neighborhoods with high-quality amenities, which could eventually lead to a decrease in feelings of loneliness.

Furthermore, a direct relation was found between loneliness and neighborhood attachment. Older adults who had higher feelings of loneliness were less likely to feel attached to their neighborhood. This relation was also confirmed by previous studies [32,46,58]. Thus, neighborhood activities could be organized and attractive meeting spaces (e.g., in a community center or park) could be developed that support neighborhood attachment and social interactions among neighbors, which could help to increase the social network size and reduce feelings of loneliness. 

Although previous studies found a direct relation between social satisfaction and/or loneliness [11,24] and urban density, this study did not find a direct or indirect relation. More in-depth research is needed on the actual relationships between urban density and loneliness.

Next, the results of this study showed that satisfaction with local amenities was related to self-perceived health and ADL, which was subsequently related to mobility. Previous studies found a direct relation between health and loneliness [26,36,37]. In addition, Van den Berg et al. [25] found that more mobile older adults had social interactions at a larger variety of locations/amenities (e.g., public space/park, community center/church or shop/services) than less mobile older adults. Policy makers that want to support healthy aging in place should strive for a large variety of high-quality local amenities that are also good, accessible, and attractive for older adults with poorer health conditions. 

One of the limitations of this research is that it was based on a convenience sample of older adults in neighborhoods within the West Brabant region in the Netherlands. Therefore, it is not possible to generalize the outcomes to other regions in and outside the Netherlands. For future research, it would be interesting to analyze feelings of loneliness and the relation with the living environment in other regions and countries to find (cultural) differences. Future research could also include other variables of the living environment, such as detailed urban design features or walkability of the neighborhood, which might influence the social behavior of older adults and their feelings of loneliness. 

Moreover, although we used a widely used and well-developed scale for measuring loneliness [49], the measurement of loneliness remains a challenge. Loneliness is a stigmatized condition, making it difficult to admit to feeling lonely [59]. Loneliness may therefore have been underreported in our study. 

## 6. Conclusions

There is a growing interest in relationships between older adults’ social network and the living environment. However, previous studies have mainly been based on bivariate analyses or included a limited number of variables. Therefore, this study contributes to existing theory by analyzing the direct and indirect influence of sociodemographics, individual mobility characteristics, social network and participation, and characteristics of the living environment on loneliness of older adults in a more comprehensive framework, using a Bayesian belief network (BBN) modeling approach. 

The results showed that safety and high-quality amenities and services in the neighborhood are essential in supporting the aging population and decreasing feelings of loneliness. In addition, social participation and the social network in the neighborhood are important for increasing neighborhood attachment and satisfaction with the social network of older adults. 

Overall, results of this study are relevant to urban planners and policy makers who focus on creating livable and healthy social neighborhoods for the aging population and preventing or reducing loneliness. Specifically, when the health of older adults decreases and subsequently their mobility and activities decrease, they become more dependent on their neighborhood.

## Figures and Tables

**Table 1:** Principal component analysis mobility intensity.

Item	Factor: Mobility Intensity
Car driver	0.705
Bicycling	0.677
Walking	0.758
Eigenvalue	1.5
% of explained variance Walking	51

**Table 2:** Principal component analysis social participation.

Item	Factor: Satisfaction with Social Network
Satisfaction social contacts	0.884
Satisfaction network size	0.889
Satisfaction network quality	0.905
Eigenvalue	2.4
% of explained variance	80

**Table 3:** Principal component analysis locations for social contacts.

Item	Factor 1: Recreation Spaces	Factor 2: Community Amenities	Factor 3: Shopping Amenities
shops daily goods			0.859
shops non-daily good			0.835
sport amenities		0.732	
community center		0.753	
cafes/restaurants		0.685	
green spaces	0.869		
walking/biking amenities	0.847		
recreational areas	0.669		
Eigenvalue	3.3	1.1	1.113
% of explained variance	41	14	

**Table 4:** Principal component analysis neighborhood attachment.

Item	Factor: Neighborhood Attachment
I’m attached to my neighborhood	0.624
I’m actively involved in my neighborhood	0.493
I like the type of people living in my neighborhood	0.780
I like the social contacts in my neighborhood	0.877
People in my neighborhood share similar values and beliefs	0.811
People in my neighborhood help each other	0.805
I feel at home in my neighborhood	0.815
Eigenvalue	4.0
% of explained variance	57

**Table 5:** Principal component analysis neighborhood safety.

Item	Factor: Neighborhood Safety
Safety during the day	0.927
Safety at night	0.927
Eigenvalue	1.7
% of explained variance	86

**Table 6:** Principal component analysis satisfaction with amenities in the neighborhood.

Item	Factor: Satisfaction with Amenities
Public transport	0.675
Care amenities	0.607
Sport amenities	0.793
Food & drink amenities	0.754
Community center	0.777
Green spaces	0.686
Walking & biking amenities	0.733
Recreational amenities	0.714
Eigenvalue	4.1
% of explained variance	52

**Table 7:** Variables used in the Bayesian network model analysis (N = 182).

Variables	Levels	%	Variables	Levels	%
**Sociodemographics**					
Gender	Male	44.0	Partner	No	60.4
	Female	56.0		Yes	39.6
Income level	Low	18.7	Perceived health	(very) Bad	21.4
	Medium	56.0		Neutral	37.9
	High	25.3		(very) Good	40.7
Age	65–70	17.0			
	71–75	22.5			
	76–80	19.2			
	81–85	23.6			
	86+	17.6			
**Mobility**					
Mobility Intensity	Very low	18.1	Activities of Daily Living	Low	21.4
	Low	18.1		Limited	20.9
	Medium	24.2		Ok	17.6
	High	18.7		Good	40.1
	Very high	20.9			
**Social Participation**					
Network size	Very small	22.5	Satisfaction Social Network	Very unsatisfied	14.8
	Small	36.3		Unsatisfied	24.2
	Medium	10.4		Neutral	14.3
	Large	11.5		Satisfied	30.2
	Very large	19.2		Very satisfied	16.5
Social contacts in green spaces	Very few	20.3	Social contacts in sport amenities	Very few	19.8
	Few	19.8		Few	16.5
	Medium	19.2		Medium	23.6
	Some	20.9		Some	20.3
	A lot	19.8		A lot	19.8
Social contacts in shops	Very few	19.8			
	Few	20.3			
	Medium	19.8			
	Some	20.3			
	A lot	19.8			
**Living Environment**					
Urban density	Urban	45.1	Neighborhood Safety	Very unsafe	15.4
	Suburban	19.8		Unsafe	7.1
	Rural	35.2		Safe	36.3
				Very safe	41.2
Distance to green	Very small	19.8	Distance to shops	Very small	18.1
	Small	39.6		Small	34.6
	Large	21.4		Medium	7.1
	Very large	19.2		Large	22.5
				Very large	17.6
Neighborhood attachment	Very unattached	23.6	Satisfaction with amenities	Very unsatisfied	28.0
	Unattached	16.5		Unsatisfied	12.1
	Neutral	18.1		Neutral	20.3
	Attached	23.1		Satisfied	15.9
	Very attached	18.7		Very satisfied	23.6
**Loneliness**					
Feelings of loneliness	Not lonely	23.1			
	Hardly lonely	25.3			
	Little bit lonely	12.6			
	Lonely	23.1			
	Very lonely	15.9			

**Table 8:** Updated probabilities (in %) for feelings of loneliness based on level of satisfaction with social network.

Satisfaction Social Network	Feelings of Loneliness					Total
	Not Lonely	Hardly Lonely	Little Bit Lonely	Lonely	Very Lonely	
Very unsatisfied	0	7	7	30	56	100
Unsatisfied	2	14	14	43	27	100
Neutral	12	31	3	23	4	100
Satisfied	40	36	9	13	2	100
Very satisfied	53	33	7	7	0	100
No evidence	23	25	13	22	16	100

**Table 9:** Updated probabilities (in %) for neighborhood attachment based on feelings of loneliness.

Feelings of Loneliness	Neighborhood Attachment					Total
	Very Unattached	Unattached	Neutral	Attached	Very Attached	
Not lonely	8	15	18	25	34	100
Hardly lonely	18	6	38	18	20	100
Little bit lonely	7	41	6	21	25	100
Lonely	31	24	13	20	12	100
Very lonely	55	12	8	12	14	100
No evidence	23	17	19	20	21	100

**Table 10:** Updated probabilities (in %) for satisfaction with amenities based on activities of daily living (ADL).

ADL	Satisfaction with Amenities					Total
	Very Unsatisfied	Unsatisfied	Neutral	Satisfied	Very Satisfied	
Low	49	14	12	5	20	100
Limited	33	16	22	23	6	100
Ok	37	11	10	11	31	100
Good	14	12	25	19	31	100
No evidence	27	14	20	16	23	100

**Table 11:** Updated probabilities (in %) for neighborhood attachment based on neighborhood safety.

Safety	Neighborhood Attachment					Total
	Very Unattached	Unattached	Neutral	Attached	Very Attached	
Very unsafe	37	15	21	12	14	100
Unsafe	28	19	18	20	15	100
Safe	21	18	21	23	17	100
Very safe	18	17	16	20	28	100
No evidence	23	17	19	20	21	100

**Table 12:** Updated probabilities (in %) for satisfaction with social network based on neighborhood safety.

Safety	Satisfaction Social Network					Total
	Very Unsatisfied	Unsatisfied	Very Unsatisfied	Unsatisfied	Very Unsatisfied	
Very unsafe	22	25	11	28	13	100
Unsafe	15	21	25	24	15	100
Safe	23	19	18	27	13	100
Very safe	11	22	14	25	29	100
No evidence	17	21	16	26	20	100

## References

[1] United Nations (2017). World Population Prospects: The 2017 Revision, Key Findings and Advance Tables.

[2] Ritsema van Eck J., Van Dam F., De Groot C., De Jong A. (2013). Demografische Ontwikkelingen 2010–2040. Ruimtelijke Effecten en Regionale Diversiteit.

[3] Patterson I. (2002). Baby boomers and adventure tourism: The importance of marketing the leisure experience. World Leis..

[4] Pino L., González-Vélez A.E., Prieto-Flores M.-E., Ayala A., Fernandez-Mayoralas G., Rojo-Perez F., Martinez-Martin P., Forjaz M.J. (2013). Self-perceived health and quality of life by activity status in community-dwelling older adults. Geriatr. Gerontol. Int..

[5] Tang F., Lee Y. (2011). Social support networks and expectations for aging in place and moving. Res. Aging.

[6] Von Hippel W., Henry J.D., Matovic D. (2008). Aging and social satisfaction: Offsetting positive and negative effects. Psychol. Aging.

[7] Cattan M., White M., Bond J., Learmouth A. (2005). Preventing social isolation and loneliness among older people: A systematic review of health promotion interventions. Ageing Soc..

[8] Ettema D., Gärling T., Olsson L.E., Friman M. (2010). Out-of-home activities, daily travel, and subjective well-being. Transp. Res. A Policy Pract..

[9] Helliwell J.F., Putnam R.D. (2004). The social context of well-being. Philos. Trans. R. Soc. Lond. B Biol. Sci..

[10] Schwanen T., Wang D. (2014). Well-being, context, and everyday activities in space and time. Ann. Am. Assoc. Geogr..

[11] Delmelle E.C., Haslauer E., Prinz T. (2013). Social satisfaction, commuting and neighborhoods. J. Transp. Geogr..

[12] Umberson D., Montez J.K. (2010). Social relationships and health: A flashpoint for health policy. J. Health Soc. Behav..

[13] Boldy D., Grenade L., Lewin G., Karol E., Burton E. (2010). Older peoples decisions regarding ‘ageing in place’: A Western Australian case study. Australasian J. Ageing.

[14] World Health Organization (2007). Global Age-Friendly Cities: A Guide.

[15] Davey J., Nana G., De Joux V., Arcus M., NewZealand Institute for Research on Ageing/Business & Economic Research Ltd. (2004). Accommodation Options for Older People in Aotearoa/New Zealand.

[16] Greenfield E.A. (2015). Support from neighbors and aging in place: Can NORC programs make a difference?. Gerontologist.

[17] Keenan T.A. (2010). Home and Community Preferences of the 45+ Population.

[18] Levasseur M., Généreux M., Bruneau J.-F., Vanasse A., Chabot É., Beaulac C., Bédard M.-M. (2015). Importance of proximity to resources, social support, transportation and neighborhood security for mobility and social participation in older adults: Results from a scoping study. BMC Public Health.

[19] Dallago L., Perkins D.D., Santinello M., Boyce W., Molcho M., Morgan A. (2009). Adolescent place attachment, social capital, and perceived safety: A comparison of 13 countries. Am. J. Community Psychol..

[20] Livingston M., Bailey N., Kearns A. (2008). People’s Attachment to Place: The Influence of Neighbourhood Deprivation.

[21] Bowling A., Stafford M. (2007). How do objective and subjective assessments of neighbourhood influence social and physical functioning in older age? Findings from a British survey of ageing. Sos. Sci. Med..

[22] Weijs-Perrée M., Van den Berg P., Arentze T., Kemperman A. (2017). Social networks, social satisfaction and place attachment in the neighborhood. Region.

[23] Perissinotto C.M., Cenzer I.S., Covinsky K.E. (2012). Loneliness in older persons: A predictor of functional decline and death. JAMA Intern. Med..

[24] Scharf T., De Jong-Gierveld J. (2008). Loneliness in urban neighbourhoods: An Anglo-Dutch comparison. Eur. J. Ageing.

[25] Van den Berg P., Kemperman A., De Kleijn B., Borgers A. (2016). Ageing and loneliness: The role of mobility and the built environment. Trav. Behav. Soc..

[26] De Jong-Gierveld J., Van Tilburg T. (2010). The De Jong Gierveld short scales for emotional and social loneliness: Tested on data from 7 countries in the UN generations and gender surveys. Eur. J. Ageing.

[27] De Jong-Gierveld J. (1984). Eenzaamheid: Een Meersporig Onderzoek.

[28] Demakakos P., Nunn S., Nazroo J., Banks J., Breeze E., Lessof C., Nazroo J. (2006). Loneliness, relative deprivation and life satisfaction. Retirement, Health and Relationships of the Older Population in England: The 2004 English Longitudinal Study of Ageing (Wave 2).

[29] Moorer P., Suurmeijer T.P.B.M. (2001). The effects of neighbourhoods on size of social network of the elderly and loneliness: A multilevel approach. Urban Stud..

[30] Wenger G.C. (1997). Social networks and the prediction of elderly people at risk. Aging Ment. Health..

[31] Wenger G.C., Tucker I. (2002). Using network variation in practice: Identification of support network type. Health Soc. Care Community.

[32] Weijs-Perrée M., Van den Berg P., Arentze T., Kemperman A. (2015). Factors influencing social satisfaction and loneliness: A path analysis. J. Transp. Geogr..

[33] Bonsang E., Soest A. (2010). Satisfaction with job and income among older individuals across European countries. SSRN Elec. J..

[34] Wrzus C., Hänel M., Wagner J., Neyer F.J. (2013). Social network changes and life events across the life span: A meta-analysis. Psychol. Bull..

[35] Pinquart M., Sörensen S. (2001). Influences on loneliness in older adults: A meta-analysis. Basic Appl. Soc. Psychol..

[36] Coyle C.E., Dugan E. (2012). Social isolation, loneliness and health among older adults. J. Aging Health.

[37] Havens B., Hall M. (2001). Social isolation, loneliness, and the health of older adults. Indian J. Gerontol..

[38] Kweon B.-S., Sullivan W.C., Wiley A.R. (1998). Green common spaces and the social integration of inner-city older adults. Environ. Behav..

[39] Metz D. (2000). Mobility of older people and their quality of life. Transp. Policy.

[40] Spinney J.E., Scott D.M., Newbold K.B. (2009). Transport mobility benefits and quality of life: A time-use perspective of elderly Canadians. Transp. Policy.

[41] Glanz T.A. (2011). Walkability, Social Interaction, and Neighborhood Design. Regional Planning Program: Student Projects and Theses. http://digitalcommons.unl.edu/cgi/viewcontent.cgi?article=1005&context=arch_crp_theses.

[42] Hacihasanoğlu R., Yildirim A., Karakurt P. (2012). Loneliness in elderly individuals, level of dependence in activities of daily living (ADL) and influential factors. Arch. Gerontol. Geriatr..

[43] Nyqvist F., Victor C.R., Forsman A.K., Cattan M. (2016). The association between social capital and loneliness in different age groups: A population-based study in Western Finland. BMC Public Health.

[44] Van der Houwen K., Kloosterman R. (2011). Vertrouwen in En Contacten met Buurtgenoten. https://www.cbs.nl/nl-nl/achtergrond/2011/13/veel-vertrouwen-en-contact-in-de-buurt.

[45] Roh S., Jang Y., Chiriboga D.A., Kwag K.H., Cho S., Bernstein K. (2011). Perceived neighborhood environment affecting physical and mental health: A study with Korean American older adults in New York City. J. Immigr. Minor. Health.

[46] Kearns A., Whitley E., Tannahill C., Ellaway A. (2015). ‘Lonesome town’? Is loneliness associated with the residential environment, including housing and neighborhood factors?. J. Community Psychol..

[47] Suurmeijer T.P., Doeglas D.M., Moum T., Briançon S., Krol B., Sanderman R., Guillemin F., Bjelle A., Van den Heuvel W.J. (1994). The Groningen activity restriction scale for measuring disability: Its utility in international comparisons. Am. J. Public Health.

[48] Hendriks S. (2009). Woonzorgzones. Woonzorgzones en de Woontevredenheid vna 75-Plussers. Een Onderzoek naar Woonzorgzone Drielanden in Harderwijk.

[49] De Jong-Gierveld J., Van Tilburg T. (2006). A 6-item scale for overall, emotional, and social loneliness. Res. Aging.

[50] Arentze T.A., Timmermans H.J.P. (2009). Regimes in social-cultural events-driven activity sequences: Modeling approach and empirical application. Transp. Res. A Policy Pract..

[51] Heckerman D., Mandani A., Wellman M.P. (1995). Real-world applications of Bayesian networks. Commun. ACM.

[52] Pearl J. (1988). Probabilistic Reasoning in Intelligent Systems: Networks of Plausible Inference.

[53] Kemperman A.D.A.M., Timmermans H.J.P. (2014). Green spaces in the direct living environment and social contacts of the aging population. Landsc. Urban. Plan..

[54] Cheng J., Bell D., Liu W. (2002). Learning Bayesian networks from data: An information-theory based approach. Artif. Intell..

[55] Lauritzen S.L. (1995). The EM algorithm for graphical association models with missing data. Comput. Stat. Data Anal..

[56] BayesFusion (2018). Genie 2.1. https://www.bayesfusion.com/news/.

[57] Hur M., Morrow-Jones H. (2008). Factors that influence residents’ satisfaction with neighborhoods. Environ. Behav..

[58] Afshar P.F., Foroughan M., Vedadhir A., Tabatabaei M.G. (2016). The effects of place attachment on social well-being in older adults. Educ. Gerontol..

[59] Morrison P.S., Smith R., Sagan O., Miller E. (2017). Loneliness: An overview. Narratives of Loneliness: Multidisciplinary Perspectives from the 21st Century.

